# Effects of different pelvic osteotomy surgeries on acetabular center and pelvic morphology

**DOI:** 10.1186/s13018-023-04062-3

**Published:** 2023-08-04

**Authors:** Can Liu, Kongjian Wang, Zhongwen Tang, Jie Wen, Sheng Xiao

**Affiliations:** 1 Department of Anatomy, Hunan Normal University School of Medicine, Changsha, 410013 Hunan China; 2 Department of Pediatric Orthopedics, Hunan Provincial People’s Hospital, The First Affiliated Hospital of Hunan Normal University, Changsha, 410005 Hunan China; 3 Department of Pediatric Orthopedics, Changde First Hospital of Traditional Chinese Medicine, Changde, 415000 Hunan China

**Keywords:** Salter pelvic osteotomy, Pemberton pelvic osteotomy, Triple pelvic osteotomy, Children's hip joint disease, Center of acetabulum, Pelvic morphology

## Abstract

**Objective:**

To compare the effects of Salter pelvic osteotomy, Pemberton pelvic osteotomy, and triple pelvic osteotomy on the center of acetabulum and pelvic morphology in children with hip joint disease.

**Methods:**

The data of children treated with Salter pelvic osteotomy (2 males and 14 females with an average age of 2.49 years), Pemberton pelvic osteotomy (4 males and 11 females with an average age of 6.11 years), and triple pelvic osteotomy(4 males and 8 females with an average age of 9.59 years) between January 2011 and December 2020 were collected. After discharge, the outpatient review was followed up for at least 1 year. All patients underwent anterior–posterior pelvic X-ray scanning before surgery, three months after surgery in the first year and every six months after the first year. The following X-ray features were analyzed: bilateral pelvic height (PH), iliac crest inclination (ICI), a horizontal distance of the acetabulum center (HD), and vertical distance of the acetabulum center (VD).

**Results:**

The mean follow-up time was 16.9 ± 4.9 months in the Salter group, 20.7 ± 5.1 months in the Pemberton group, and 18.0 ± 5.4 months in the triple group (all P > 0.05). No significant differences between PH, HD, and VD of both sides on the preoperative AP pelvic x-ray were found. However, at the last follow-up, PH, HD,VD, and ICI increased in the Salter group (all P < 0.05), PH and VD increased in the Pemberton group (all P < 0.05), and VD decreased in the Triple group (P < 0.05).

**Conclusion:**

Salter pelvic osteotomy may cause pelvic height to increase and the center of acetabulum to move outward and downward. In contrast, Pemberton pelvic osteotomy may cause pelvic height to increase and the center of acetabulum to move downward. Triple pelvic osteotomy only causes the center of acetabulum to move downward.

## Background

Hip joint disease in children is a rare condition affecting the hip joint and all the healthy activities children usually engage in. Common childhood hip joint diseases include developmental dysplasia (DDH) and Perthes disease. DDH is the most common abnormality in newborn infants, with a prevalence of 1–2/1000 and 5–30/1000 in an unscreened and surveyed population, respectively [1]. Perthes disease is commonly caused by **idiopathic avascular necrosis** of proximal femoral epiphysis, and its prevalence rate is 0.4–29/100,000 [2]. Both conditions may cause hip pain, limited mobility, and lameness. If conservative treatment fails or the condition is not timely diagnosed, a pelvic osteotomy is usually required to delay the hip-joint degeneration and reduce the incidence of early hip arthritis. Osteotomy can increase the inclusion of the femoral head and achieve hip reduction. So far, different pelvic osteotomy methods have been used to treat DDH, Perthes, and other diseases with good results. Widely used methods include Salter pelvic osteotomy, Pemberton pelvic periacetabular osteotomy, triple osteotomy, and other osteotomy methods, which are chosen based on the patient's age, whether the Y-shaped cartilage is closed, whether the pubic symphysis is fused, the state of femoral head dislocation, and the development of the acetabulum and femoral head [3].

Salter pelvic osteotomy was first proposed by Salter RB in 1961 [4]. He used the pubic symphysis as a hinge to change the acetabulum through the rotation of the distal end of the osteotomy direction to increase the inclusion of the anterolateral femoral head [5, 6]. Salter pelvic osteotomy is generally suitable for children with DDH who are between 18 months and six years old, and it can generally reduce the acetabular index by 15°–20° [7–9].

Pemberton first proposed his osteotomy in 1965[10]. This procedure is suitable for 1–12-year-old children with hip dislocation whose acetabular index needs to be corrected by more than 15°, a shallow and steep acetabulum, and a small head of the acetabulum. Osteotomy starts between the anterior iliac spines and extends posteriorly to above the Y-shaped cartilage, keeping the posterior side of the ilium continuous, using the Y-shaped cartilage as a hinge, and correcting the acetabulum by controlling different parts of the posterior medial cortex of the ilium osteotomy. Regarding biomechanics, Pemberton osteotomy seems to have a lower effect on pelvic biomechanics than Salter osteotomy [11]. However, Pemberton osteotomy reduces the volume of the acetabulum, has a more significant hip load than average [12], and causes the risk of premature closure of the Y-shaped cartilaginous epiphysis [13].

Steel first proposed the surgical method of triple pelvic osteotomy in 1971 [14], which was further improved by Tönnis in 1983 [15]. In recent years, triple pelvic osteotomy has become the preferred method for treating adolescent acetabular dysplasia and other hip joint diseases [16]. The acetabular osteotomy block has greater mobility and rotation to improve femoral head containment. Although the amputation of the ilium, pubis, and ischium increases the mobility of the acetabular fragments, it causes discontinuity of the pelvis and instability of the acetabular fragments [15], requiring strong internal fixation and plaster immobilization. In addition, a spherical confluence is more likely to occur after triple osteotomy than in other pelvic osteotomies [16].

However, all these methods may also lead to complications, such as avascular necrosis of the femoral head and restricted hip joint mobility, stiffness, and re-dislocation. Also, few studies reported changes in the morphology of the pelvis.

In this study, three different pelvic osteotomies, including Salter pelvic osteotomy, Pemberton periacetabular pelvic osteotomy, and triple osteotomy, were compared before and after surgery, and the center of acetabulum and pelvic height were compared between the operative side and the non-operative side to check the differences of pelvic morphology between three different pelvic osteotomies. Because of the different methods and effects of various pelvic osteotomies in children, we compared the bilateral pelvic height, iliac crest inclination, the horizontal distance of the hip joint rotation center, and acetabular rotation on the anteroposterior pelvic film before and at the last follow-up in the three groups of children patients. Center-to-center vertical distances were used to assess changes in the center of acetabular rotation and pelvic morphology.

## Methods

### General Information

Children who underwent unilateral pelvic osteotomy in our department between January 2011 and December 2020 were included in the study. Inclusion criteria were the following: (1) children with hip diseases (limited to DDH and Perthes disease) who received only unilateral Salter pelvis osteotomy, Pemberton pelvis osteotomy, or triple pelvis osteotomy; (2) no previous pelvic surgery for other reasons; (3) with no bone tumor, bone tuberculosis, and other diseases; (4) follow-up time > one year.

### Surgical methods

After general anesthesia, the patient was placed in a supine position, and all three pelvic osteotomies were performed using the same groin incision. After the inner and outer wall of the ilium was subperiosteally exposed, the muscles in the patient diagnosed with DDH were retracted to reveal the hip capsule. Consequently, hip capsulotomy was performed, the ligamentum teres was resected, and the fibrofatty pulvinar was cleaned out. There was no need to open a hip capsule for the patient diagnosed with Perthes disease. Subperiosteal dissection of the inner and outer table of the ilium extended to the sciatic notch level and was done with a sharp periosteal elevator in all patients.

Salter osteotomy was performed from the sciatic notch to 1–2 mm proximal to the anterior inferior iliac spine (AIIS). The distal fragment was pulled distally to allow for anterior and caudal rotation of the acetabulum through the symphysis, and an autogenous iliac wedge was inserted in the middle, fixed with 2–3 Kirschner wires.

Pemberton osteotomy was performed toward the ilioischial limb of the triradiate cartilage, which ended between the notch and the posterior portion of the acetabulum. The distal end of the osteotomy was overturned downward, and an autogenous iliac wedge was inserted in the middle.

Triple osteotomy added a vertical incision in the genitofemoral fold used to finish the osteotomy of the pubis and Ischium. The superior ramus was approached extraperiosteally. Two Hohmann retractors were placed, protecting the soft tissues during the osteotomy. The osteotomies were carried out near the pubis with an osteotome. The inferior ramus was accessed by bluntly dissecting through the adductor magnus. Iliac osteotomy was performed using the same groin incision from the sciatic notch to just above the anteroinferior iliac spine. The acetabulum block was pushed through the pubic and iliac incisions and was rotated downward, outward, and forward. Two Kirschner wires were used for fixation from the proximal end to the distal end, and one Kirschner wire was used for fixation from the distal end to the proximal end.

After surgery, all the children were treated with a spica cast fixation for six weeks, the cast was removed six weeks later, and the hip joint weight-free functional exercise was performed for two weeks before walking. In addition, pelvic orthography was reviewed six weeks after surgery, and every three months thereafter.

### Parameters measurement

**Fig. 1:**
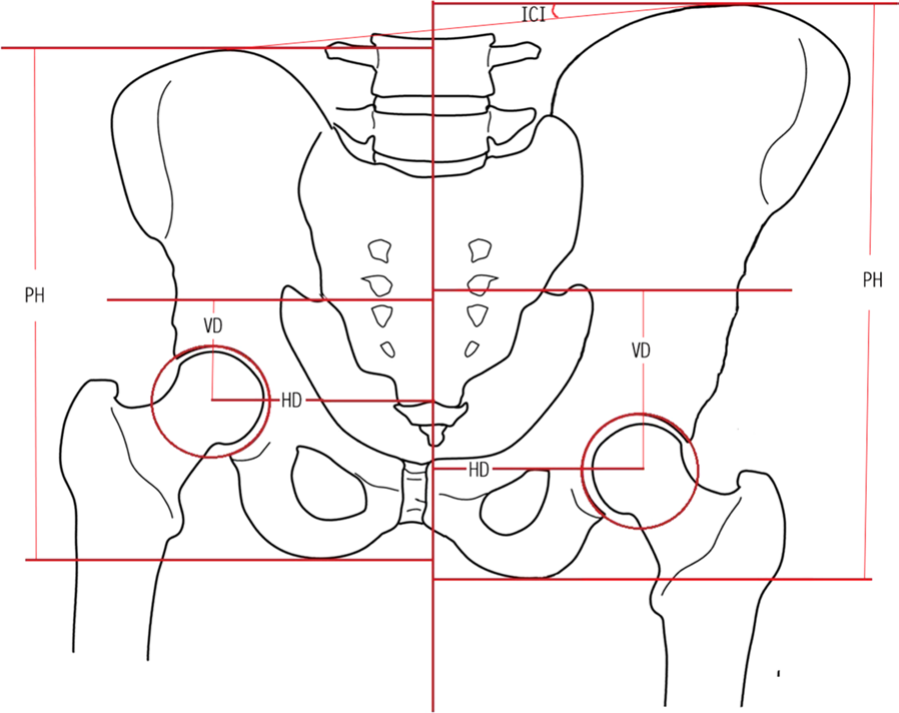
The parameters measured on pevic X-ray. Iliac crest inclination (ICI): the angle between the line connecting the top of the iliac crest and the teardrop line (defined as the higher of the operative side than the non-operative side as positive, and the lower of the operative side than the non-operative side as negative); Pelvis height (PH): Make a parallel line between the highest point of the ilium and the lowest point of the ischium, the vertical distance between the two parallel lines. Horizontal distance of acetabulum rotation center(HD): the vertical distance between the acetabulum rotation center and the midline of sacrum. Vertical distance of acetabulum center (VD): the distance from the acetabulum center to lowest level of sacroiliac joint (the lowest line of sacroiliac joint vertical to the midline of sacrum)

After discharge, the outpatient review was followed up for at least 1 year. All patients underwent X-ray scanning every three months in the first year and every six months after one year. The following X-ray features were analyzed (Fig. 1): (1) iliac crest inclination (ICI): the angle between the line connecting the top of the iliac crest and the teardrop line (it was defined as positive if the value on operative side higher than the non-operative, and negative if the operative side lower than the non-operative side); (2) pelvis height (PH): a parallel line was drawn between the highest point of the ilium and the lowest point of the ischium, the vertical distance between the two parallel lines; (3) horizontal distance of acetabulum rotation center(HD): the vertical distance between the acetabulum rotation center and the midline of sacrum; (4) vertical distance of acetabulum center (VD): the distance from the center of acetabulum to the lowest level of the sacroiliac joint (the lowest line of the sacroiliac joint vertical to the midline of sacrum).

Positive post-preoperative differences were defined as the distance on the operative side being larger than the distance on the non-operative side. Otherwise, they were recorded as negative. The percentage difference was calculated as (operative − non-operative)/non-operative *100%.

### Statistical analysis

SPSS26.0 statistical software was used for analysis. Kruskal–Wallis H test was used to compare the sex ratio, operative age, and follow-up time of different osteotomy methods. Normality test and homogeneity of variance test were performed to compare the pelvic height, horizontal distance, and vertical distance of the acetabulum center on the operative side and non-operative side. Wilcoxon signed-rank test was used if significant differences were found.

Kruskal–Wallis and Npar tests were performed to compare preoperative and post-operative iliac crest inclination, a percentage difference in pelvic height, a percentage difference in horizontal distance between the center of acetabulum, and a percentage difference in vertical distance between the two centers of acetabulum. Wilcoxon signed-rank test was used for statically significant differences.

## Results

A total of 16 children (2 males and 14 females) were treated with Salter pelvic osteotomy with an average age of 2.49 years; 15 children (4 males and 11 females) were treated with Pemberton pelvic osteotomy with an average age of 6.11 years; 12 children, 4 males and 8 females, underwent triple osteotomy with an average age of 9.59 year old (P < 0.001 for all 3 groups).

All patients were followed up for more than 12 months. The mean follow-up time was 16.9 ± 4.9(12–31) months in the Salter pelvic osteotomy group, 20.7 ± 5.1 (15–33)months in the Pemberton periacetabular osteotomy group, and 18.0 ± 5.4(12–31) months in the Triple osteotomy group (Table 1); there was no significant difference between groups (all P > 0.05).

**Table 1:** General data of three different pelvic osteotomies (using Kruskal–Wallis H test)

	Salter	Pemberton	Triple	P-value
No	16	15	12	
Gender (girl:boy)	2:14	4:11	4:8	
Age of surgery (YO)	2.49	6.11	9.59	< 0.001
Follow-up time (months) DDH/LCPDOP OP side (L/R)	16.9 ± 4.9(12–31) 13/3 10/6	20.7 ± 5.1(15–33) 13/2 9/6	18.0 ± 5.4(12–31) 10/2 7/5	0.537 < 0.001

YO: Year old; DDH: Developmental dysplasia of hip; LCPD: Legg–Calve–Perthes disease; OP: Operation

There were three cases of wound problems in the three groups, with no important blood vessel and nerve injury; 1 case in the Salter group had local pain; no patient in the Pemberton group had local pain; 2 cases in the triple group had local pain. After analgesic treatment, the pain was relieved in all three cases.

**Fig. 2:**
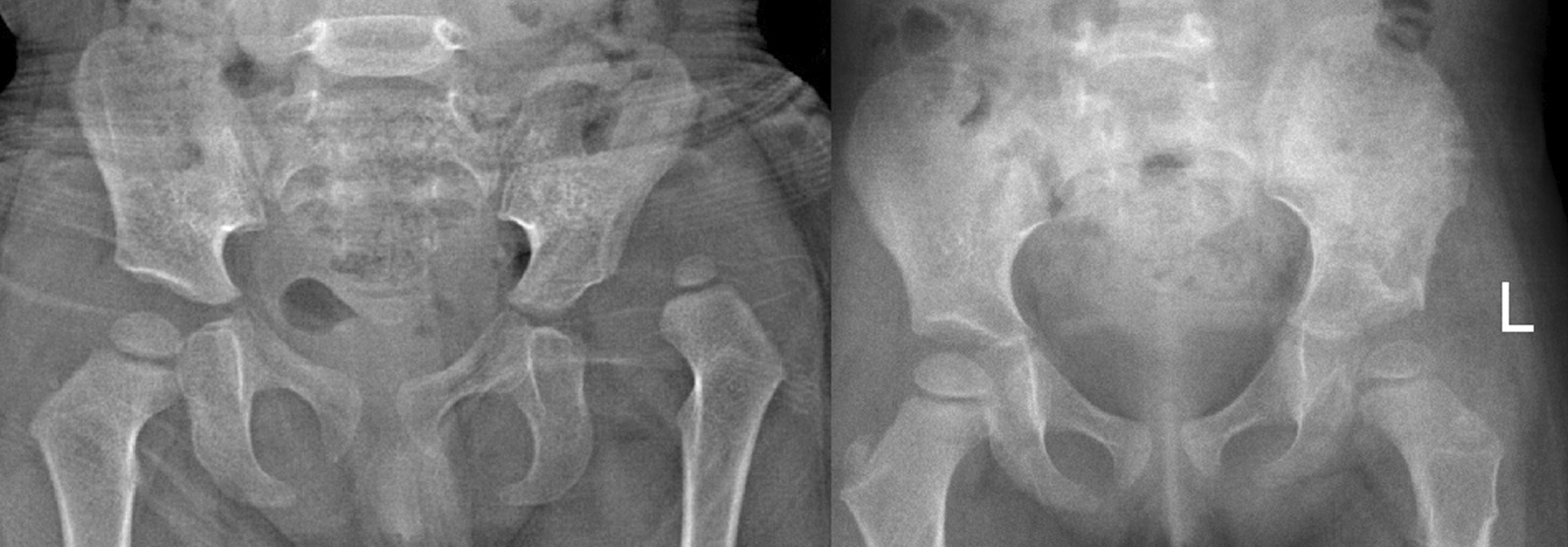
Typical case in Salter group, 3 years old, 2-year follow-up shows PH, VD, HD increase

On the preoperative pelvic anteroposterior radiograph, the height, the hip joint rotation center, and the vertical distance of the acetabulum center of affected pelvis were the same as those of the contralateral side (Table 2). At the last follow-up, in the Salter group, the average PH on the operative side was 136.22 mm compared to 126.95 on the non-operative side (P < 0.05), the average HD on the operative side was 64.46 mm compared to 56.93 on the non-operative side (P < 0.05), and the average HD on the operative side was 53.32 mm compared to 43.86 on the non-operative side (P < 0.05) (Table 3). The mean ICI increased by 1.94% compared with the preoperative mean (P < 0.05); the percentage of the mean PH difference increased by 21.10% (P < 0.05), the percentage difference of the average HD increased by 30.52% (P < 0.05); the average percentage of the VD decreased by 51.20% (P < 0.05) (Fig. 2).

**Table 2:** Comparison of the operative side and the non-operative side of different pelvic osteotomies before surgery (using paired-sample Wilcoxon signed-rank test)

Preoperative	Operative side	Non-operative side	P-value
Salter group			
PH (mm)	112.84	112.87	0.877
HD (mm)	49.71	49.64	0.796
VD (mm)	35.78	35.63	0.469
Pemberton group			
PH (mm) HD (mm) VD (mm)	145.99 60.34 43.58	146.63 60.39 43.67	0.460 0.955 0.913
Triple group			
PH (mm) HD (mm) VD (mm)	185.29 74.33 62.02	185.21 74.81 62.19	0.638 0.158 0.307

**Table 3:** Comparison of the operative side and the non-operative side of different pelvic osteotomies after surgery (using paired-sample Wilcoxon signed-rank test)

Postoperative	Operative side (50th percentile)	Non-operative side (50th percentile)	P-value
Salter group			
PH (mm) HD (mm) VD (mm)	136.22 64.46 53.32	126.95 56.93 43.86	0.000 0.000 0.000
Pemberton group			
PH (mm) HD (mm) VD (mm)	167.82 69.66 56.27	159.16 69.39 50.23	0.001 0.776 0.001
Triple pelvic osteotomy			
PH (mm) HD (mm) VD (mm)	206.05 84.93 75.82	203.07 84.19 67.75	0.060 0.433 0.003

**Fig. 3:**
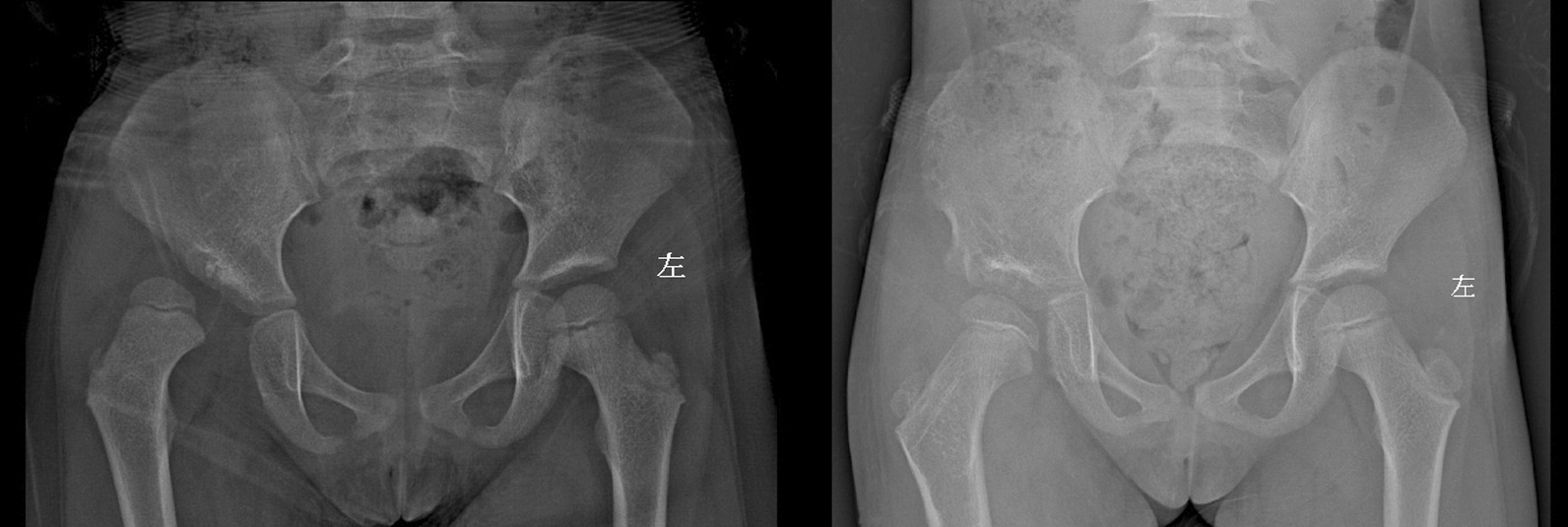
Typical case in Pemberton group, 5 years old, 2-year follow-up shows VD increase

At the last follow-up in the Pemberton group, the average PH on the operative side was 167.82 mm compared to 159.16 on the non-operative side (P < 0.05); the average HD on the operative side was 69.66 mm compared to 69.39 on the non-operative side (P > 0.05); the average VD on the operative side was 56.27 mm compared to 50.23 on the non-operative side (P < 0.05). The mean ICI increased by 1.94% compared with the preoperative mean (P < 0.05); the percentage of the mean PH difference increased by 15.50% (P < 0.05), the percentage of the mean HD difference increased by 15.97% (P > 0.05), and the percentage of the mean VD difference increased by 15.97% (P < 0.05) (Fig. 3).

**Fig. 4:**
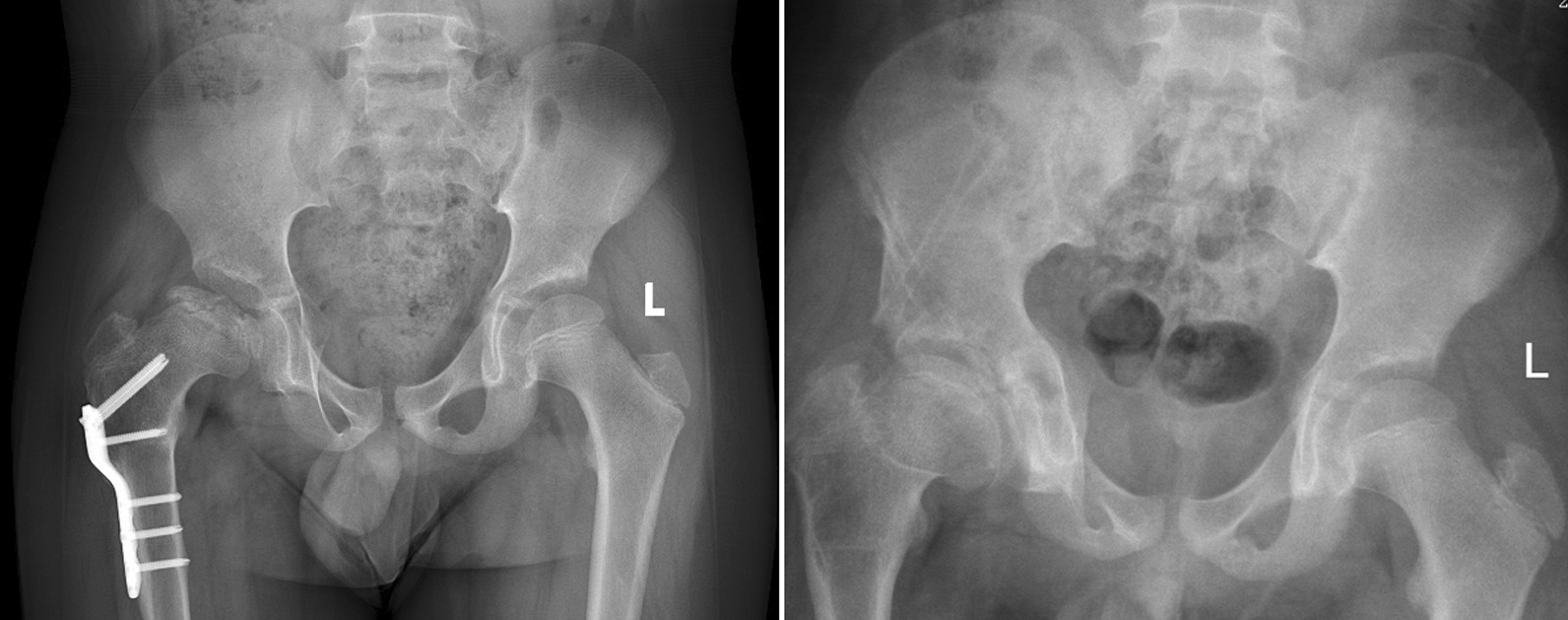
Typical case in triple group, 10 years old, this patient was diagnosed as Perthes disease, received femoral varus osteotomy in 8 years old, 2-year follow-up shows VD increase

At the last follow-up in the triple group, the average PH on the operative side was 206.05 mm compared to 203.07 on the non-operative side (P > 0.05); the average HD on the operative side was84.93 mm compared to 84.19 on the non-operative side (P > 0.05); the average VD on the operative side was 75.82 mm compared to 67.75 on the non-operative side (P < 0.05). The mean ICI increased by 0.08% compared with the preoperative mean (P > 0.05); the percentage of the mean PH difference increased by 11.35% (P > 0.05), the percentage of the mean HD difference increased by 15.32% (P > 0.05), the percentage of the mean VD difference increased by 22.81% (P < 0.05) (Fig. 4).

According to the above-mentioned result, Salter pelvic osteotomy may cause pelvic height to increase and the center of acetabulum to move outward and downward. In contrast, Pemberton pelvic osteotomy may cause pelvic height to increase and the center of acetabulum to move downward. Triple pelvic osteotomy causes the center of acetabulum to move downward.

## Discussion

Since introduction of pelvic osteotomy, studies have been mainly focusing on reducing the related complications caused by pelvic osteotomies, such as hip stiffness, redislocation, epiphyseal changes, femoral osteonecrosis, and changes in pelvic biomechanics [9, 11, 17], while changes in pelvic morphological structure post-surgery have been rarely reported in their study. Wang et al*.* found no difference in functional outcomes and pelvic imbalance in children with DDH who underwent Salter osteotomy and Pemberton (and were followed-up for more than ten years) [18]. Yet, comparisons of pelvic morphology in the shorter term were not available for at least five years, and they did not perform short-term comparisons of preoperative and postoperative, operative versus non-operative pelvic heights for a single surgical modality. However, anthropological evidence shows a direct relationship between hip function and pelvic morphology [19]. Masanori et al. revealed that DDH patients have structural abnormalities in the pelvis [20]. The abnormal acetabular morphology is caused by local dysplasia around the hip joint and is affected by the morphological characteristics of the whole pelvis. In addition, children are in the stage of rapid growth and development. Abnormal pelvic morphology in the short term after surgery may severely impact the functional development of their hip joints. Therefore, early (after six weeks) rehabilitation training is crucial for restoring hip function in children.

In this study, we found no significant difference in the pelvis height on both sides before surgery in three pelvic osteotomies. However, after Salter pelvic osteotomy, the pelvic height of the operative side was higher than that of the non-operative side and the iliac crest inclination. The percentage of pelvic height difference was also significantly higher than before the operation. This difference manifests in pelvic tilt and lengthening, compensating the sacroiliac joints and even the spine, resulting in complications such as scoliosis, abnormal posture, and unequal length of the lower limbs [18, 21, 22]. Pemberton osteotomy can achieve the same morphologic change in pelvic lengthening without pelvic tilt. This outcome after Salter pelvic osteotomy and Pemberton periacetabular osteotomy may occur because both surgical methods require the insertion of a wedge-shaped osteotomy from the osteotomy end [4, 10], resulting in Pelvic bone healing with pelvic elevation. There was no significant difference in the pelvic height between the operative and non-operative sides after triple pelvic osteotomy and iliac crest inclination difference before and after surgery (Table 4). This may be because the bone craft rotate center in triple pelvic osteotomy is the center of acetabulum, which is different from the pubic symphysis in Salter pelvic osteotomy and triradiate cartilage in Pemberton pelvic osteotomy [14]. Therefore, there were no changes in pelvic height and iliac crest inclination after triple pelvic osteotomy, or corresponding compensation of the spine and lower extremities.

**Table 4:** Preoperative and postoperative comparison of different pelvic osteotomies (using paired-sample Wilcoxon signed-rank test)

	Post-preoperative (%)
Salter group	
ICI (°) Percentage increase in PH (%) Percentage difference in HD (%) Percentage difference in VD (%)	1.94% 21.10% 30.52% 51.20%
Pemberton group	
ICI (°) Percentage increase in PH (%) Percentage difference in HD (%) Percentage difference in VD (%)	0.20% 15.50% 15.97% 38.57%
Triple group	
ICI (°) Percentage increase in PH ( %) Percentage difference in HD (%) Percentage difference in VD (%)	0.08 11.35% 15.32% 22.81%

Currently, for the center of acetabulum, most scholars focus on the acetabular cup placement during total hip replacement [23–25]. However, few scholars reported on the displacement of the acetabulum center after pelvic osteotomy. The change in the position of the center of acetabular rotation leads to the change in the moment arm of the lower limbs, which in turn leads to the change in the gluteal muscle length and balance control of children [26, 27]. Herein, we found there is no significant difference in the center of acetabular rotation preoperatively between children with Salter osteotomy, Pemberton periacetabular osteotomy, and triple pelvic osteotomy. After the Salter osteotomy, the horizontal and vertical distance of the acetabulum center on the operative side was larger than that on the non-operative side, indicating that the center of acetabular rotation shifted laterally and downward after Salter pelvic osteotomy. Yet in the Pemberton group, there was only a vertical distance of the acetabulum center increase on the operative side, which can also be explained by the different bone craft rotation centers in two pelvic osteotomies; in Salter pelvic osteotomy, the bone craft rotated circle was centered in pubic symphysis, while in Pemberton pelvic osteotomy, the bone craft rotated circle was centered in triradiate cartilage. In their study, Naci et al*.* revealed that Pemberton periacetabular osteotomy had much less impact on pelvic biomechanics than Salter osteotomy [11].

Moreover, we also found that after triple pelvic osteotomy, the vertical distance of the acetabulum center on the operative side was smaller than that on the non-operative side, which suggests that the triple pelvic osteotomy will result in a downward shift in the center of the acetabular rotation. When the rotation center of the acetabulum moves down, the center of rotation of the femoral head moves down to a certain extent, stretches the abductor and adductor muscles, increases muscle tension, and affects the biomechanics of the hip joint, which is not consistent with Hsu’s results, who proposed an improved pelvic triple osteotomy (MTI osteotomy) and found that the femoral head would be displaced laterally after surgery [28]. The difference between our study and that of Hsu is that our patients were children, and the triradiate cartilage was open, thus having greater pelvic remodel potential.

Studies have shown that children with DDH have not only hip dysplasia but also problems with the morphology of the pelvis and the central position of the original acetabulum [20, 29, 30]. Previous studies [19,31,32] disregarded the overall morphology of the pelvis and the center of acetabular rotation. Spine-pelvis-lower extremities as a whole, elongation and inclination of the pelvis can lead to sacral inclination leading to scoliosis and lower extremity compensation [18, 21, 22]. Since pelvic osteotomy will lead to changes in the morphology of the pelvis and the movement of the center of acetabular rotation, focus should be placed on the changes in the original center of rotation of the acetabulum and the pelvic morphology during pelvic osteotomy and try not to change the morphology of the pelvis. It is necessary to try to get the center of acetabulum close to the original center of acetabulum to prevent spine and lower extremity compensation to the greatest extent and restore the biomechanics of the hip joint in children with DDH.

This study has a few limitations. This was a retrospective study with a small sample size which may lead to bias. Prospective cohort studies with large samples are needed to further verify the reported results.

## Conclusion

In summary, different pelvic osteotomies bring different changes on center of acetabulum and pelvic height, which could help the orthopedist to choose pelvic osteotomies according to the pelvic situation before osteotomy and adequately predict the prognosis after osteotomy.

## References

[1] Zhang S, Doudoulakis KJ, Khurwal A (2020). Developmental dysplasia of the hip. Br J Hosp Med (Lond).

[2] Yagdiran A, Zarghooni K, Semler JO (2020). Hip pain in children. Dtsch Arztebl Int.

[3] Venkatadass K, Durga Prasad V, Al Ahmadi NMM, Rajasekaran S (2022). Pelvic osteotomies in hip dysplasia: why, when and how?. EFORT Open Rev.

[4] Salter RB, Dubos JP (1974). The first fifteen year's personal experience with innominate osteotomy in the treatment of congenital dislocation and subluxation of the hip. Clin Orthop Relat Res.

[5] Selberg CM, Chidsey B, Skelton A (2020). Pelvic osteotomies in the child and young adult hip: indications and surgical technique. J Am Acad Orthop Surg.

[6] Schulze A, Tingart M (2016). Salter innominate osteotomy: Indications, surgical technique, results. Orthopade.

[7] Utterback JD, MacEwen GD (1974). Comparison of pelvic osteotomies for the surgical correction of the congenital hip. Clin Orthop Relat Res.

[8] Wong-Chung J, Ryan M, O'Brien TM (1990). Movement of the femoral head after Salter osteotomy for acetabular dysplasia. J Bone Joint Surg Br.

[9] Tejpal T, Shanmugaraj A, Gupta A (2020). Outcomes and complications of patients undergoing Salter's innominate osteotomies for hip dysplasia: a systematic review of comparative studies. J Hip Preserv Surg.

[10] Pemberton PA (1965). Pericapsular osteotomy of the ilium for treatment of congenital subluxation and dislocation of the hip. J Bone Joint Surg Am.

[11] Ezirmik N, Yildiz K (2012). A biomechanical comparison between salter innominate osteotomy and pemberton pericapsular osteotomy. Eurasian J Med.

[12] Chang CF, Wang TM, Wang JH (2011). Adolescents after Pemberton's osteotomy for developmental dysplasia of the hip displayed greater joint loading than healthy controls in affected and unaffected limbs during gait. J Orthop Res.

[13] Plaster RL, Schoenecker PL, Capelli AM (1991). Premature closure of the triradiate cartilage: a potential complication of pericapsular acetabuloplasty. J Pediatr Orthop.

[14] Steel HH (1973). Triple osteotomy of the innominate bone. J Bone Joint Surg Am.

[15] Tönnis D, Behrens K, Tscharani F (1981). A modified technique of the triple pelvic osteotomy: early results. J Pediatr Orthop.

[16] Vukasinović Z, Spasovski D, Zivković Z (2009). Triple pelvic osteotomy in the treatment of hip dysplasia. Sep Arh Celok Lek.

[17] Wu KW, Wang TM, Huang SC (2010). Analysis of Osteonecrosis following Pemberton acetabuloplasty in developmental dysplasia of the hip: long-term results. J Bone Joint Surg Am.

[18] Wang CW, Wang TM, Wu KW (2016). The comparative, long-term effect of the Salter osteotomy and Pemberton acetabuloplasty on pelvic height, scoliosis and functional outcome. Bone Joint J..

[19] Hogervorst T, Bouma H, de Boer SF (2011). Human hip impingement morphology: an evolutionary explanation. J Bone Joint Surg Br.

[20] Fujii M, Nakashima Y, Sato T (2011). Pelvic deformity influences acetabular version and coverage in hip dysplasia. Clin Orthop Relat Res.

[21] Yu Y, Song K, Wu B, et al. (2021) Coronal compensation mechanism of pelvic obliquity in patients with developmental dysplasia of the hip. Global Spine J. 21925682211010760. 10.1177/21925682211010760 PMC10189343 33949240

[22] Barry K, McManus F, O'Brien T (1992). Leg lengthening by the transiliac method. J Bone Joint Surg Br.

[23] Asayama I, Chamnongkich S, Simpson KJ (2005). Reconstructed hip joint position and abductor muscle strength after total hip arthroplasty. J Arthroplasty.

[24] D'Lima DD, Chen PC, Colwell CW (2001). Optimizing acetabular component position to minimize impingement and reduce contact stress. J Bone Joint Surg Am..

[25] Yoder SA, Brand RA, Pedersen DR (1988). Total hip acetabular component position affects component loosening rates. Clin Orthop Relat Res.

[26] Wu T, Lohse KR, Van Dillen L, Song K, Clohisy JC, Harris MD. Are abnormal muscle biomechanics and patient-reported outcomes associated in patients with hip dysplasia? Clin Orthop Relat Res. 2023. 10.1097/CORR.0000000000002728 PMC10642886 37289006

[27] Stirling P, Viamont-Guerra MR, Strom L, Chen AF, Saffarini M, Nover L, Laude F (2021). Does cup position at the high hip center or anatomic hip center in THA for developmental dysplasia of the hip result in better harris hip scores and revision incidence? A systematic review. Clin Orthop Relat Res.

[28] Hsu JY, Lee CC, Lin SC, et al. Radiographic outcomes of ganz versus modified triple osteotomies in femoral head medialization and coverage in acetabular dysplasia. J Clin Med. 2022;11(7). 10.3390/jcm11071924 PMC9000010 35407532

[29] Tannast M, Pfannebecker P, Schwab JM (2012). Pelvic morphology differs in rotation and obliquity between developmental dysplasia of the hip and retroversion. Clin Orthop Relat Res.

[30] Tanaka T, Moro T, Ishikura H, Hashikura K, Kaneko T, Tanaka S (2021). Characteristics of three-dimensional acetabular morphology of patients with excellent outcome after rotational acetabular osteotomy over 20 years. J Orthop Surg Res.

[31] Ertürk C, Altay MA, Işikan UE (2013). A radiological comparison of Salter and Pemberton osteotomies to improve acetabular deformations in developmental dysplasia of the hip. J Pediatr Orthop B.

[32] Bursali A, Tonbul M (2008). How are outcomes affected by combining the Pemberton and Salter osteotomies?. Clin Orthop Relat Res.

